# Identification of QTLs for resistance to maize rough dwarf disease using two connected RIL populations in maize

**DOI:** 10.1371/journal.pone.0226700

**Published:** 2019-12-17

**Authors:** Xintao Wang, Qing Yang, Ziju Dai, Yan Wang, Yingying Zhang, Baoquan Li, Wenming Zhao, Junjie Hao

**Affiliations:** 1 Crop Designing Center, Henan Academy of Agricultural Sciences, Zhengzhou, China; 2 Institute of Food Crops, Jiangsu Academy of Agricultural Sciences, Nanjing, China; 3 Plant Protection Institute, Henan Academy of Agricultural Sciences, Zhengzhou, China; Huazhong Agriculture University, CHINA

## Abstract

Maize rough dwarf disease (MRDD) is a significant viral disease caused by rice black-streaked dwarf virus (RBSDV) in China, which results in 30% yield losses in affected summer maize-growing areas. In this study, two connected recombinant inbred line (RIL) populations were constructed to elucidate the genetic basis of resistance during two crop seasons. Ten quantitative trait loci (QTLs) for resistance to MRDD were detected in the two RILs. Individual QTLs accounted for 4.97–23.37% of the phenotypic variance explained (PVE). The resistance QTL (*qZD-MRDD8-1*) with the largest effect was located in chromosome bin 8.03, representing 16.27–23.37% of the PVE across two environments. Interestingly, one pair of common significant QTLs was located in the similar region on chromosome 4 in both populations, accounting for 7.11–9.01% of the PVE in Zheng58×D863F (RIL-ZD) and 9.43–13.06% in Zheng58×ZS301 (RIL-ZZ). A total of five QTLs for MRDD resistance trait showed significant QTL-by-Environment interactions (QEI). Two candidate genes associated with resistance (*GDSL-lipase* and *RPP13-like* gene) which were higher expressed in resistant inbred line D863F than in susceptible inbred line Zheng58, were located in the physical intervals of the major QTLs on chromosomes 4 and 8, respectively. The identified QTLs will be studied further for application in marker-assisted breeding in maize genetic improvement of MRDD resistance.

## Introduction

Maize (*Zea mays* L.) is an important food crop worldwide and is a major global source of protein and carbohydrates for humans and livestock. However, maize is the natural host of several viruses, some of which cause diseases that affect yield and quality in some maize production zones [1, 2]. In China, maize rough dwarf disease (MRDD) was first discovered in Xinjiang and Gansu in 1954, and then spread to other provinces [3, 4]. The main symptoms of MRDD include internode shortening, malformed tassels and significant delays in vegetative growth [5–7]. Four virus species in the genus *Fijivirus*, Mal de Río Cuarto virus (MRCV), maize rough dwarf virus (MRDV), rice black-streaked dwarf virus (RBSDV) and southern rice black-streaked dwarf virus (SRBSDV), are the causal pathogens of MRDD. MRDD is mainly caused by brown planthoppers carrying RBSDV in China [3, 4, 8].

No pesticides are available to cure RBSDV-infected maize [9]. Thus, MRDD resistance breeding is the most effective and environmentally friendly strategy for disease control [10, 11]. No genotypes with complete resistance to MRDD have ever been reported but a few highly resistant lines have been identified in China, most of which are mainly derived from P78599 (a US hybrid) [12, 13].

In recent years, some studies have been conducted to detect QTLs for MRDD resistance in maize, and a number of resistance QTLs distributed on all maize chromosomes have been identified [14–20]. For example, Wang et al. [10] reported that resistance to MRDD is a quantitative trait that involves small individual effects. Di Renzo et al. [14] found two QTLs (chromosome bins 1.03 and 8.03) for resistance to Mal de Río Cuarto disease using a BLS14 × Mo17 population with 227 F_2:3_ families and 56 SSR markers. Additionally, using F_2:3_ populations and recombinant inbred lines (RILs) derived from 90110 × Ye478 crosses, Wang et al. [15] and Luan et al. [16] identified three and five QTLs for resistance to MRDD, respectively. A major QTL was located on chromosome 8 in these two populations. Moreover, the resistance QTL on chromosome 8.03 was consistently identified in two independent studies [17, 18], and the effect of this QTL was confirmed with a genome-wide association study (GWAS) by Chen et al. [19]. Recently, using SLAF-seq and SSR analysis, a major MRDD resistance locus on chromosome 6 was detected in F_2_ populations derived from a cross between the resistant line K36 and susceptible line S221 [20].

Most of the QTLs for MRDD resistance have been located at different chromosomal sites in diverse populations, suggesting that the inconsistent results in the above reports need to be further tested and validated [21–27]. In this study, two maize RIL populations with a common female parent were used to detect QTLs for resistance to MRDD during two crop seasons. The major objectives of this study were to: (1) further reveal the genetic basis of resistance to MRDD in two connected RILs, (2) identify and characterize the QTL for MRDD resistance in these populations by inclusive and genome-wide composite interval mapping (ICIM and GCIM) approach, and (3) find consistent or major QTL for further fine mapping and marker-assisted breeding. These results will contribute to a better understanding of the genetic basis of resistance to MRDD in maize.

## Materials and methods

### Genetic materials

Two sets of RIL populations were used. One maize F_8_ RIL consisted of 241 lines derived from a cross between the Zheng58 and D863F lines. The second RIL population consisted of 242 F_7_ RILs derived from a cross between Zheng58 and ZS301. The two sets of RILs were developed by the single-seed descent method. The common parent (Zheng58) is one of the parental lines of the hybrid Zhengdan 958, an elite hybrid planted broadly in China. D863F and ZS301 were obtained from the Chinese Academy of Agricultural Sciences.

### Evaluation of MRDD resistance in the field

The two populations and three parents were evaluated for MRDD resistance under natural infection over two years (2016 and 2017) in a high incidence area for MRDD: Kaifeng (N34°47′, E114°20′) in Henan Province. At the flowering stage, all plants were evaluated and scored for resistance to MRDD based on a disease rating scale of 0–4. The rating 0 indicates no symptom and 4 represents a highly susceptible phenotype [28, 29]. The disease severity index (DSI) of each RIL was calculated using the following equation: DSI (%) = ∑(disease rating scale score × number of plants in rating) × 100/(4 × total number of plants) [30].

### Genotyping

Young leaves were collected from both RIL populations and their parents, and then genomic DNA for genotyping was isolated using a CTAB method [31]. In this study, 1,890 simple sequence repeat (SSR) primer pairs from the MaizeGDB (http://www.maizegdb.org/) and newly developed primers (S1 Table) were used to screen for polymorphisms between the two parental lines of each RIL population. Approximately 220 primer pairs showed polymorphisms between Zheng58 and D863F, and 190 primer pairs showed polymorphisms between Zheng58 and ZS301. Of these, 215 and 181 markers were used to construct genetic linkage maps in RIL-ZD and RIL-ZZ, respectively. The amplified PCR products were examined using a 3500xl DNA Analyzer (Applied Biosystems, Foster City, CA, USA). The reaction volume was 10 μl, consisting of 0.1 μl Taq DNA polymerase, 0.15 μl 10 mmol L^−1^ dNTP, 1.6 μl 10× PCR buffer (Mg^2+^), 0.4 μl forward, reverse and M13 primers, 2 μl DNA template, and 5.75 μl water.

### Linkage map construction and QTL analysis

Genetic linkage maps were constructed from the SSR genotypes in both RILs using the JoinMap software version 4.0. To maximize the accuracy of mapping data, QTL IciMapping 4.0 based on a inclusive composite interval mapping (ICIM) model and QTL.gCIMapping via genome-wide composite interval mapping (GCIM) method were used to detect the QTL [32–34]. A joint analysis of the two RIL populations was performed using the joint inclusive composite interval mapping (JICIM) method in IciMapping 4.0 [35]. QTL×environment interaction effects (QEI) in two connected RIL populations were estimated for MRDD resistance using the QTL IciMapping 4.0 [36].

### Phenotypic data analysis

Phenotypic data were subjected to analysis of variance using the software package SPSS 12.0 (Chicago, IL, USA). The broad-sense heritability (*h*^2^) of MRDD resistance in each environment was calculated as follows: $h^{2}=\sigma_{g}^{2}/(\sigma_{g}^{2}+\sigma_{gy}^{2}/n+\sigma_{e}^{2}/nr)$, where $\sigma_{g}^{2}$ represents the genotype variance, $\sigma_{gy}^{2}$ represents the genotype-by-environment interaction variance, $\sigma_{e}^{2}$ represents the error variance, and *n* and *r* are the number of environments and replications per environment, respectively.

### Quantitative real-time PCR

The plants of Zheng58 and D863F were sown in 15 cm pots (three plants per pot) in a growth chamber under LD condition (15 h light and 9 h dark). The seedlings at the third leaf stage were exposed for 3 d in inoculation chambers with brown planthoppers. The leaves at 0 h, 24 h and 72 h after treatment were harvested, immediately frozen in liquid nitrogen and stored at –80°C. Three biological replicates, each consisting of leaves from three plants per sample, were performed.

Total RNA was isolated using TRIzol reagent (Invitrogen, Carlsbad, CA, USA) followed by a treatment with RNase-free DNase (TaKaRa, Dalian, China) according to the manufacturer’s instructions. Complementary DNA was reverse-transcribed from 2 μg of total RNA using PrimeScript^™^ 1st Strand cDNA Synthesis Kit (TaKaRa) following the manufacturer's instructions. Quantitative real-time PCR (qPCR) was performed on a Bio-Rad real-time PCR detection system (Bio-Rad, Hercules, CA, USA). The reaction volume was 15 μl, consisting of SYBR Premix Ex Taq, 7.5 μl; gene-specific primers (forward; reverse), 0.6 μl for a final 20 pmol/L concentration; water, 5.3 μl and cDNA template, 1 μl. Each reaction was performed in triplicate.

The constitutive gene *glyceraldehyde-3-phosphate dehydrogenase*(*GAPDH*) was used as endogenous control to normalize expression in maize leaves. The quantification of gene expression levels was calculated relative to *GAPDH* using a 2 –^ΔΔCT^ method [37]. The primers for the qPCR assay for each candidate gene and *GAPDH* were designed using the Primer5.0 software (S2 Table).

## Results

### Phenotypic analysis of the parental lines and two RIL populations

**Fig 1:**
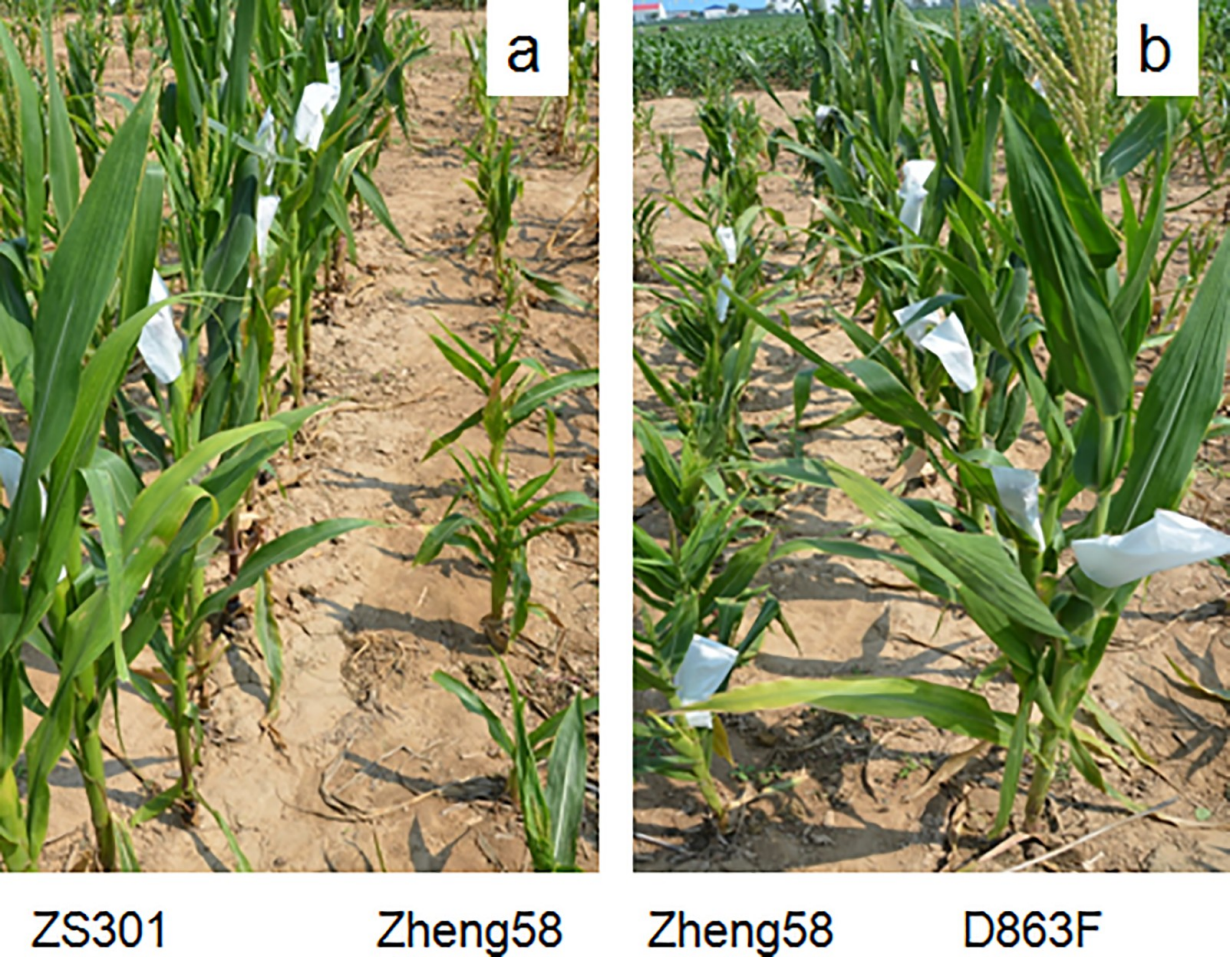
The phenotypes of RBSDV-inoculated maize. (a) The RIL-ZZ parental lines ZS301 and Zheng58 during the flowering stage. (b) The RIL-ZD parental lines Zheng58 and D863F during the flowering stage.

The performance of the three parents used to develop the RIL populations was evaluated in 2016 and 2017 (Table 1). The parental lines D863F, ZS301, and Zheng58 exhibited significant differences for resistance to MRDD under natural infection over the two years (Fig 1). Among the three parental lines, D863F was most resistant to MRDD (2016 DSI: 2.82%, 2017 DSI: 4.20%), followed by ZS301 (2016 DSI: 23.55%, 2017 DSI: 18.75%) and Zheng58 (2016 DSI: 90.25%, 2017 DSI: 93.96%). As shown in Table 1, the 241 RIL-ZD lines and 242 RIL-ZZ lines were also characterized by high variation in DSI, which ranged from 3.85 to 100% in RIL-ZD and 15.28 to 100% in RIL-ZZ. The heritability of MRDD resistance in RIL-ZD and RIL-ZZ was 0.82 and 0.87, respectively.

**Table 1:** General statistics and heritability for DSI in the two RIL populations and their parents.

Population Years		Parents		RILs					F value			H^2^(%)
		Zheng58	D863F/ ZS301	Mean±SD	Range	CV%	Skewness	Kurtosis	Genotype	Environment	GEI	
RIL-ZD	2016	90.25±7.16	2.82±0.86	71.24±16.67	22.50–100	23.40	-0.88	0.89	4.69**	24.25**	1.11**	0.82
	2017	93.96±9.23	4.20±1.68	67.23±18.84	3.85–100	28.02	-0.69	1.20				
RIL-ZZ	2016	90.25±7.16	23.55±4.86	78.26±12.70	25.00–100	16.22	-0.52	0.79	7.33**	32.42**	3.48**	0.87
	2017	93.96±9.23	18.75±2.72	62.18±12.18	15.28–100	19.59	-1.08	3.46				

**Significant at P < 0.01, SD: standard deviation. CV(%): efficient of variation. GEI: Environment × Genotype interaction. H^2^(%): broad sense heritability.

### Linkage maps

A total of 1890 SSR markers were screened, and 215 and 181 were polymorphic between the Zheng58/D863F and Zheng58/ZS301 parental genotypes. The total lengths of the linkage maps for RIL-ZD and RIL-ZZ were 1832.35 cM and 1968.48 cM, respectively. The average interval lengths were 8.52 cM (RIL-ZD) and 10.87 cM (RIL-ZZ).

### QTL identification for resistance to MRDD in the RIL-ZD population

Five QTLs for MRDD resistance were found in the RIL-ZD population by ICIM method (Table 2). The resistance QTLs were located on chromosomes 2 (bin 2.07–2.08), 4 (bin 4.07–4.08), 7 (bin 7.03–7.05), and 8 (bin 8.03 and bin 8.05). These QTLs were derived from the resistant inbred line D863F. The resistance QTL (*qZD-MRDD8-1*) with the largest effect was located in chromosome bin 8.03, linked with and flanked by SSR markers M105 and M108, representing 16.27–21.88% of the phenotypic variance explained (PVE) across the two years (Table 2). The second largest-effect QTL for MRDD resistance, *qZD-MRDD7*, which accounted for 8.48–10.73% of the PVE in the two environments, was located between SSR markers umc1841 and umc2333 in chromosome bin 7.03–7.05. The other three QTLs identified, *qZD-MRDD2* on chromosome 2, *qZD-MRDD4-1* on chromosome 4 and *qZD-MRDD8-2* on chromosome 8, represented 6.08–8.93%, 7.11–7.21% and 10.05% of the PVE, respectively.

**Table 2:** QTLs for MRDD resistance detected in the RIL-ZD population in a 2-year experiment.

	Methods	QTL	Chromosome	Marker Interval	Bin locus	Position (Mp)^a^	LOD^b^	R^2^ (%)^c^	A^d^
2016	ICIM	qZD-MRDD2	2	bnlg1316-bnlg2077	2.07–2.08	202.37–213.03	5.47	8.93	-4.94
		qZD-MRDD4-1	4	umc1620-bnlg2162	4.07–4.08	178.30–185.56	5.29	7.21	-4.47
		qZD-MRDD7	7	umc1841-umc2333	7.03–7.05	143.39–173.84	6.13	10.73	-5.64
		qZD-MRDD8-1	8	M105- M108	8.03	107.61–110.64	14.29	21.88	-10.42
		qZD-MRDD8-2	8	bnlg1599- umc1141	8.05	133.87–160.62	6.09	10.05	-5.24
	GCIM	qZD-MRDD2	2	bnlg1316-bnlg2077	2.07–2.08	202.37–213.03	4.34	8.70	4.96
		qZD-MRDD4-1	4	umc1620-bnlg2162	4.07–4.08	178.30–185.56	5.68	9.01	-6.45
		qZD-MRDD6	6	umc1656-bnlg2191	6.02	89.29–93.37	3.83	7.06	-5.51
		qZD-MRDD7	7	umc1841-umc2333	7.03–7.05	143.39–173.84	4.54	7.17	-5.34
		qZD-MRDD8-1	8	M105- M108	8.03	107.61–110.64	15.63	23.16	-11.65
		qZD-MRDD8-2	8	bnlg1599- umc1141	8.05	133.87–160.62	5.14	9.58	-6.92
2017	ICIM	qZD-MRDD2	2	bnlg1316-bnlg2077	2.07–2.08	202.37–213.03	4.09	6.08	-5.38
		qZD-MRDD4-1	4	umc1620-bnlg2162	4.07–4.08	178.30–185.56	3.12	7.11	-6.17
		qZD-MRDD7	7	umc1841-umc2333	7.03–7.05	143.39–173.84	3.09	8.48	-4.40
		qZD-MRDD8-1	8	M105- M108	8.03	107.61–110.64	11.11	16.27	-8.43
	GCIM	qZD-MRDD2	2	bnlg1316-bnlg2077	2.07–2.08	202.37–213.03	5.30	14.22	-9.78
		qZD-MRDD4-1	4	umc1620-bnlg2162	4.07–4.08	178.30–185.56	4.55	8.26	-8.14
		qZD-MRDD7	7	umc1841-umc2333	7.03–7.05	143.39–173.84	3.54	6.17	-5.34
		qZD-MRDD8-1	8	M105- M108	8.03	107.61–110.64	11.34	23.37	-12.85

^a^ The physical positions of the identified QTL according to B73 reference sequence V4.

^b^ logarithm of the odds

^c^ percent of phenotypic variance explained by single QTL

^d^ Additive effect of the QTL

By using QTL.gCIMapping, a total of six QTLs were detected in the RIL-ZD population(Table 2). Two on chromosome 8, and one each on chromosome 2, 4, 6 and 7, of which *qZD-MRDD8-1* located on chromosome 8 accounted for the most phenotypic variation (23.37%).

### QTL identification for resistance to MRDD in the RIL-ZZ population

Two QTLs for MRDD resistance were found in the RIL-ZZ population by ICIM method (Table 3). The resistance QTLs were located on chromosomes 2 (bin 2.02–2.03) and 4 (bin 4.08). These QTLs both came from the resistant line ZS301. The largest effect QTL (*qZZ-MRDD4*) was located in chromosome bin 4.08, linked with and flanked by SSR markers umc2285 and umc2041, accounting for 9.43–13.06% of the PVE across the two years (Table 3). The other QTL was a relatively minor QTL; *qZZ-MRDD2* was flanked by SSR markers umc1261 and umc1185 and represented 9.04% and 7.19% of the PVE in 2016 and 2017, respectively.

**Table 3:** QTLs for MRDD resistance detected in the RIL-ZZ population in a 2-year experiment.

	Methods	QTL	Chromosome	Marker Interval	Bin locus	Position (Mp)^a^	LOD^b^	R^2^ (%)^c^	A^d^
2016	ICIM	qZZ-MRDD2	2	umc1261-umc1185	2.02–2.03	13.55–20.77	2.76	9.04	-3.83
		qZZ-MRDD4	4	umc2285- umc2041	4.08	186.48–189.37	3.14	13.06	-4.95
	GCIM	qZZ-MRDD2	2	umc1261-umc1185	2.02–2.03	13.55–20.77	3.76	8.96	-4.67
		qZZ-MRDD4	4	umc2285- umc2041	4.08	186.48–189.37	4.83	12.33	-6.83
		qZZ-MRDD6	6	umc1133-umc1818	6.01–6.02	65.67–86.72	2.56	5.97	-3.57
2017	ICIM	qZZ-MRDD2	2	umc1261-umc1185	2.02–2.03	13.55–20.77	2.52	7.19	-3.65
		qZZ-MRDD4	4	umc2285- umc2041	4.08	186.48–189.37	3.07	9.43	-5.12
	GCIM	qZZ-MRDD2	2	umc1261-umc1185	2.02–2.03	13.55–20.77	3.59	7.23	-4.19
		qZZ-MRDD4	4	umc2285- umc2041	4.08	186.48–189.37	4.38	11.17	-5.73
		qZZ-MRDD10	10	umc1938- umc1246	10.03	83.46–98.01	2.83	4.97	-3.92

^a^ The physical positions of the identified QTL according to B73 reference sequence V4.

^b^ logarithm of the odds.

^c^ percent of phenotypic variance explained by single QTL.

^d^ Additive effect of the QTL.

Four QTLs for MRDD resistance were found in the RIL-ZZ population by GCIM method (Table 3). The resistance QTLs were located on chromosomes 2, 4, 6 and10. The largest effect QTL (*qZZ-MRDD4*) was located on bin 4.08, accounting for 11.17–12.33% of the PVE across two years.

### Comparison of QTLs between the two connected RILs

A total of ten QTLs (six in RIL-ZD and four in RIL-ZZ) for MRDD resistance were detected by QTL mapping in this study. Among these MRDD-resistance QTLs, one pair of QTLs was located in the similar region on chromosome 4: *qZD-MRDD4-1* in RIL-ZD and *qZZ-MRDD4* in RIL-ZZ. *qZD-MRDD4-1* and *qZZ-MRDD4* were derived from the resistant inbred lines D863F and ZS301, respectively, whereas the major QTL on chromosome 8 (*qMRDD1-8*) was only found in the RIL-ZD population.

### QTL for MRDD resistance detected by JICIM in two RIL populations

The JICIM approach detected four QTLs on chromosomes 1, 2, 4 and 8 across the two connected RIL populations in the two environments (Table 4). The MRDD resistance QTLs on chromosomes 4 (*qMRDD-4*) and 8 (*qMRDD-8*) were detected in both years, while *qMRDD-2* was only detected in 2016 and *qMRDD-1* was only detected in 2017. *qMRDD8* was located in chromosome bin 8.03, linked with and flanked by SSR markers M105 and M108, accounting for 12.32–16.03% of the PVE across the two years. *qMRDD4* was in chromosome bin 4.07–4.08 and explained 5.32–7.78% of the phenotypic variance.

**Table 4:** QTLs for MRDD resistance detected by joint inclusive composite interval mapping (JICIM) in the two RIL populations.

	QTL	Chromosome	Marker Interval	Bin locus	Position (Mp)^a^	LOD^b^	R^2^ (%)^c^	LOD-ZD^d^	LOD-ZZ^e^	A^d^-ZD^f^	A^d^-ZZ^g^
2016	qMRDD-2	2	bnlg1316-bnlg1141	2.08	213.03–218.02	4.60	6.81	4.51	0.09	-6.56	-0.54
	qMRDD-4	4	umc1620-bnlg2162	4.07–4.08	178.30–185.56	5.25	7.78	2.53	2.72	-5.16	-2.43
	qMRDD-8	8	M105- M108	8.03	107.61–110.64	8.26	12.32	8.12	0.14	-8.81	0.68
2017	qMRDD-1	1	bnlg1025-dupssr12	1.07–1.08	223.69–243.21	2.81	3.82	2.45	0.35	-4.42	-1.02
	qMRDD-4	4	umc1620-bnlg2162	4.07–4.08	178.30–185.56	4.52	5.32	2.19	2.23	-3.09	-2.62
	qMRDD-8	8	M105- M108	8.03	107.61–110.64	12.34	16.03	12.25	0.10	-10.20	0.53

^a^The physical positions of the identified QTL according to B73 reference sequence V4. logarithm of the odds.

^b^logarithm of the odds.

^c^percent of phenotypic variance explained.

^d^LOD in RIL-ZD.

^e^LOD inRIL-ZZ.

^f^Additive effect of the QTL in RIL-ZZ.

^g^Additive effect of the QTL in RIL-ZZ.

### QTL × environment interactions for MRDD resistance

In order to estimate the interaction between QTLs and environments for MRDD resistance, the QTL×environment interaction (QEI) analysis was conducted in two connected RILs (Table 5). In RIL-ZD, a total of four QEIs were identified and distributed on chromosomes 4, 8 and 10, the phenotypic variation explained by each QTL ranged from 3.35 to 6.74%, and the phenotypic variation explained by each QTL × environment interaction ranged from 0.02 to 3.63%.

In RIL-ZZ, one QEI for MRDD resistance was identified and distributed on chromosome 4, the phenotypic variation explained by QTL and QTL×environment interaction were 3.79% and 0.09%, respectively.

**Table 5:** QTL×environment interactions for resistance to MRDD in two RIL populations.

	QTL	Chromosome	Marker Interval	LOD^a^	PVE^b^	PVE(A)^c^	PVE(AE)^d^	A^d^ ^e^	AE1^f^	AE2^g^
RIL-ZD	qZD-MRDD4-1	4	umc1620-bnlg2162	4.60	3.35	3.33	0.02	-4.00	-0.28	0.28
	qZD-MRDD4-2	4	umc2288-umc2289	3.46	3.65	3.23	0.42	-3.08	1.11	-1.11
	qZD-MRDD6	6	umc1656-bnlg2191	4.47	4.61	3.93	0.69	-3.43	1.43	-1.43
	qZD-MRDD8	8	M105- M108	10.90	6.74	3.11	3.63	-3.88	4.19	-4.19
RIL-ZZ	qZZ-MRDD4	4	umc2285-umc2041	3.57	3.79	3.70	0.09	-2.23	0.34	-0.34

^a^ logarithm of the odds.

^b^ percent of phenotypic variance explained.

^c^ phenotypic variance explained by additive effect.

^d^ Phenotypic variance explained by additive QTL×environment interactions

^e^ Additive effect of the QTL

^f^ AE1 represent the additive effects of additive QTL in 2016.

^g^ AE2 represent the additive effects of additive QTL in 2017.

### Candidate gene prediction and qPCR analysis

To further explore the molecular mechanism of MRDD resistance in maize, we identified candidate genes through the National Center for Biotechnology Information (NCBI) and MaizeGDB databases. According to the BLAST results, several candidate genes associated with resistance were located in the physical intervals of the QTLs on chromosomes 4 and 8, such as *GDSL-lipase* and *RPP13-like*.

**Fig 2:**
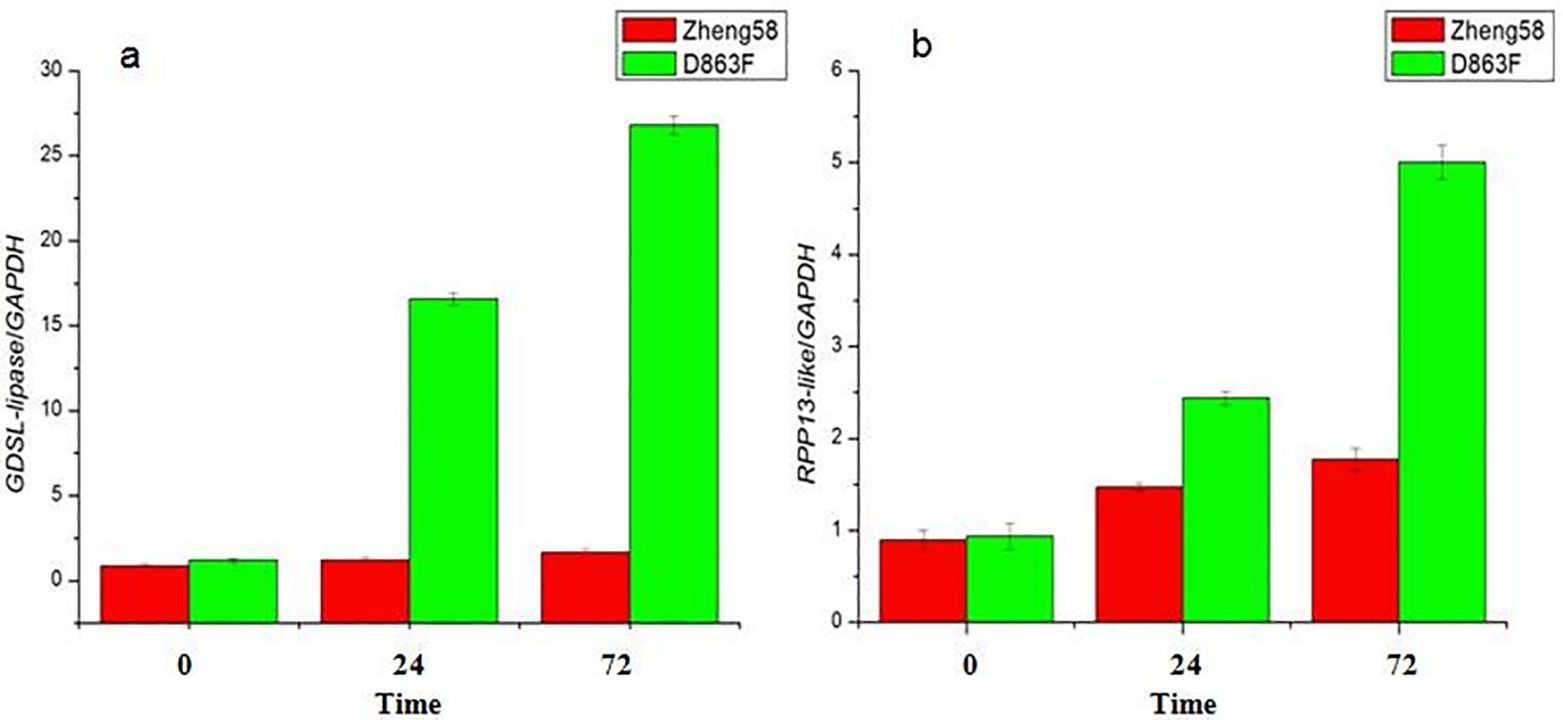
Real-time PCR quantitative analysis of two candidate genes in Zheng58 and D863F plants. (a) qPCR analysis of *GDSL-lipase*. (b) qPCR analysis of *RPP13-like*. The average values (mean ± SEM) are based on three independent experiments.

In order to further test the behavior of candidate genes, we used a qPCR assay to measure mRNA abundance in the leaves of Zheng58/D863F (Fig 2). In resistant inbred line D863F, the *GDSL-lipase* and *RPP13-like* expression began to rapidly increase after RBSDV infection, finally reaching highest level at 72 h. However, *GDSL-lipase* and *RPP13-like* expression had little change in susceptible inbred line Zheng58, and the expression level of these two genes was higher in resistant inbred line D863F than in susceptible inbred line Zheng58. The above results indicated that *GDSL-lipase* and *RPP13-like* might contribute to MRDD resistance in maize.

## Discussion

### Analyzing MRDD resistance in two connected populations

Identification of QTLs/genes conferring resistance to MRDD and resistance breeding are the most effective and economic measures for controlling this disease [20]. Thus, screening and evaluation of MRDD resistance in maize germplasm is necessary for breeding resistant maize hybrids. In our previous study, we identified some different resistant materials that might contain different resistance genes [38]. In this study, the paternal line D863F exhibited high resistance and ZS301 displayed moderate resistance, whereas the maternal line Zheng58 showed high susceptibility. The DSI results showed that the resistant lines D863F and ZS301 might each contain specific resistance genes.

### Comparison of QTLs detected in the two RIL populations

We verified six and four QTLs for MRDD resistance in RIL-ZD and RIL-ZZ in the two environments, respectively (Tables 2 and 3). Among the MRDD-resistance QTLs identified in the two connected RILs, *qZD-MRDD4-1* and *qZZ-MRDD4* were located in a similar region on chromosome 4, suggesting that these QTLs might be controlled by the same gene(s). However, several other QTLs for MRDD resistance were detected between the different RIL populations. Five QTLs (*qZD-MRDD2*, *qZD-MRDD6*, *qZD-MRDD7*, *qZD-MRDD8-1* and *qZD-MRDD8-2*) were detected in RIL-ZD, while only three minor QTLs (*qZZ-MRDD2*, *qZZ-MRDD6* and *qZZ-MRDD10*) were identified in RIL-ZZ. These results suggest that resistance to MRDD in maize might be controlled by population-specific QTLs, and *qZD-MRDD8-1* should contain an important resistance gene based on its effect on MRDD disease. ICIM is a widely used method for mapping QTLs in biparental populations [39], and GCIM was reported to have improved QTLs detection power, especially small QTLs [40]. In this study, we compared the outputs of IciMapping and gCIMapping, and found 7 common QTLs for MRDD resistance trait. Among them, QTL *qZD-MRDD8-1* was mapped in the marker interval of M105-M108 by both ICIM and GCIM approaches. It should be noted that three small QTLs (*qZD-MRDD6*, *qZZ-MRDD6*, *qZZ-MRDD10*) for MRDD resistance were detected only by gCIMapping. Based on the results of this study and previous investigations [26, 27, 41], we speculate that MRDD resistance is a complex genetic trait that is regulated by multiple resistance genes.

Plant growth and development are controlled by genetic composition and environment, as well the interaction between them [42]. In maize, in spite of that QTL×environment interaction for some traits have been described [43, 44], QTL×environment interaction for MRDD resistance has not been reported so far. In this study, five QEIs for MRDD resistance were detected for multi-environment phenotypic values using IciMapping, indicating that the genetic and molecular mechanisms of resistance to MRDD were partly influenced by the environment. However, since these QEIs effects for MRDD resistance were relatively small, it is assumed that this trait was more significantly affected by genotype than by environment.

### Comparison of QTLs detected in previous studies

To date, several labs have identified numerous QTLs conferring resistance to MRDD. A major resistance QTL has been consistently mapped in the region of bin 8.03 on chromosome 8 in different mapping populations [16, 17]. Additionally, a major QTL for MRCV resistance at the same position on chromosome 8 was identified by Di Renzo et al. [14], Bonamico et al. [23] and Rossi et al. [25]. In our study, *qZD-MRDD8-1* in RIL-ZD was located in a similar region on chromosome 8 across two years. These results suggest that resistance to different MRDD viruses might have a common mechanism in maize and this region on chromosome 8 might have a core gene homolog conferring resistance to MRDD. We strongly believe that this QTL could serve as a major QTL for gene cloning or marker-assisted selection.

A QTL (*qZD-MRDD4-1* and *qZZ-MRDD4*) on chromosome 4 was common to both RIL populations. In a previous study, Hao et al. [27] detected a locus significantly associated with MRDD DSI on chromosome 4.09 by GWAS. Bonamico et al. [23] also identified a QTL for MRCV resistance on chromosome 4.05. The genomic locations of these QTLs suggest that they are distinct from *qZD-MRDD4-1*/*qZZ-MRDD4*. Therefore, we suppose that *qZD-MRDD4-1*/*qZZ-MRDD4* is a novel and stable QTL conferring resistance to MRDD.

In recent QTL experiments, Luan et al. [16] detected a QTL for MRDD resistance on bin 2.02 through QTL mapping. Li et al. [20] detected a single dominant locus significantly associated with MRDD on bin 6.01 by SLAF-seq. Rossi et al. [23] identified a QTL for MRCV resistance on bin 10.03. In our study, *qZD-MRDD2* in RIL-ZD and *qZZ-MRDD6*/*qZZ-MRDD10* in RIL-ZZ were located in a similar region on chromosomes 2, 6 and 10 across one or two years.

### Associations between QTLs and candidate genes for MRDD resistance in maize

The virus-resistance mechanism for MRDD in maize is very complex, and little is known about the molecular mechanism and candidate genes for MRDD resistance in maize [20, 41]. According to the BLAST results, several candidate genes were located in the physical intervals of the QTLs on chromosomes 4 and 8, such as *GDSL-lipase* on chromosome 4 and *RPP13-like* on chromosome 8. *GDSL-lipase* genes are well represented in the plant immune system, and have been shown to be induced by both biotic and abiotic stress. *GDSL LIPASE1* is involved in pathogen responses in *Arabidopsis thaliana* [45]. *RPP13* is thought to function independently of NDR1/EDS1 and SA responses to *Peronospora parasitica* in *Arabidopsis* [46]. These predicted genes could be possible candidates for resistance to MRDD. Further studies are necessary to construct near isogenic lines at bins 4.07 and 8.03 and perform positional cloning.

## Supporting information

S1 Table Information on newly developed primers used in linkage map construction and QTL analysis in this study. (XLSX)

S2 Table Primer sets for real-time PCR analysis in this study. (XLSX)
